# Exploring Barriers Faced by Community Pharmacists in Serving Patients with Disabilities in Saudi Arabia: Recommendations for Enhancing Healthcare Provisions

**DOI:** 10.3390/pharmacy12050137

**Published:** 2024-09-09

**Authors:** Aisha F. Badr

**Affiliations:** Faculty of Pharmacy, King Abdulaziz University, Jeddah 21589, Saudi Arabia; afbadr@kau.edu.sa

**Keywords:** disability, patient education, community pharmacy, communication barrier

## Abstract

While several studies have explored the barriers to accessing community pharmacies for individuals with physical, hearing, visual, and learning disabilities, most have focused on the perspectives of disabled individuals. Therefore, this study aimed to examine the barriers faced by community pharmacists when serving individuals with physical, visual, and hearing disabilities in Saudi Arabia, with the goal of recommending ways to enhance future healthcare provisions. Methods: A mixed-methods cross-sectional phenomenological study was conducted among community pharmacists in Saudi Arabia between March and April 2022. Both closed- and open-ended questions were utilized to identify themes related to community pharmacists’ barriers and experiences when providing care to individuals with disabilities. Results: A total of 40 community pharmacists participated in this study. Among them, 57.5% reported encountering difficulties when caring for patients with disabilities, with 65% indicating a lack of specialized services at their respective community pharmacies. Three major themes emerged from the findings: the need for pharmacist training and awareness, technology-guided methods for overcoming communication barriers, and improving overall pharmacy accessibility. Conclusion: This study reveals a significant gap in the provision of services for patients with disabilities in Saudi Arabia. Addressing physical accessibility, providing targeted training, and leveraging technology can enhance care delivery and promote inclusivity. Further research is warranted to assess the effectiveness of technological solutions and the integration of artificial intelligence in improving communication and patient-centered care for individuals with disabilities.

## 1. Introduction

In Saudi Arabia, community pharmacists play a vital role in healthcare, often serving as the first point of contact for many patients. Serving patients with disabilities poses unique challenges that need to be addressed to ensure that equitable and inclusive healthcare services are maintained [1]. The World Health Organization (WHO) defines disability as an umbrella term that encompasses impairments, activity limitations, and participation restrictions. Impairments refer to problems with body function or structure; activity limitations are difficulties an individual may have in executing tasks or actions; and participation restrictions are problems experienced in life situations [2].

According to the World Health Organization report on disability, it is estimated that 15% of the world population live with disabilities, with 110 (2.2%) to 190 million (3.8%) experiencing substantial difficulty in functioning. In Saudi Arabia, a survey conducted by the General Authority for Statistics estimated that over half a million Saudi citizens (1 in every 30 persons) reported some form of disability, with a higher prevalence among males and the elderly (60 years and above) [3,4].

People with disabilities have largely been overlooked as a population for public health-related attention, but recent efforts have made the poor health of this population visible [5,6,7,8,9]. Health disparities and/or inequity lead largely to unmet clinical requirements, a lack of emphasis on health promotion, and insufficient access to quality healthcare and preventative services [10].

Community pharmacists are often the health system point of entry for most patients due to extended opening hours, proximity, accessibility, and affordability, with no need for prior appointments or referrals [11]. Previous studies in Saudi Arabia have reported that Saudi healthcare consumers frequently visit their local community pharmacy for multiple reasons, including disease-related advice and purchasing over-the-counter (OTC) products, cosmetics, and prescription medicines [12,13,14,15]. Any unmet needs for this entry point can jeopardize patient care, leading to medication errors and increased overall cost [5].

People with disabilities often face significant barriers to accessing community pharmacy services, exacerbating health disparities. For instance, wheelchair users may encounter physical barriers such as steps or narrow aisles in pharmacies, making it difficult or impossible to enter or navigate the premises [2]. Individuals with visual impairments might struggle to read medication labels or locate specific products due to the lack of accessible labeling and signage [16]. Those with hearing impairments could find it challenging to communicate effectively with pharmacists, especially if pharmacies lack staff trained in sign language or do not provide alternative communication methods [17]. Moreover, individuals with intellectual or cognitive disabilities may struggle to understand medication instructions, leading to potential misuse. This issue is compounded by a lack of tailored education and support services within community pharmacies [18,19].

Although some studies discussed barriers in accessing community pharmacies in this population, the majority have focused on the perspective of the disabled person [6,8,9,16]. Only one Ethiopian study has explored pharmacists’ views and recommendations, and another Turkish study examined pharmacists’ opinions on community pharmacy accessibility for physically disabled patients [7,20].

Therefore, this study aims to critically examine key barriers encountered by community pharmacists in Saudi Arabia when providing care to patients with disabilities, shedding light on the existing challenges and potential areas for improvement.

## 2. Materials and Methods

### 2.1. Study Design

Following Institutional Review Board (IRB) approval (reference number: PH-1443-51), a mixed cross-sectional phenomenological qualitative study was conducted in Saudi Arabia, targeting community pharmacists from 13 March 2022 to 8 April 2022. The mixed-methods approach combines quantitative data, which provides a broad understanding of the prevalence and types of barriers encountered, with qualitative insights that delve deeply into the lived experiences and perceptions of the pharmacists involved.

### 2.2. Sampling Method

Convenient sampling was used by distributing an anonymized electronic survey link (google form^®^) in pharmacists’ WhatsApp groups across different regions of Saudi Arabia.

### 2.3. Data Collection

A combination of closed- and open-ended questions, along with Likert-scale items, was used to assess community pharmacists’ barriers and experiences when caring for individuals with disabilities. The questionnaire items were adapted from previous studies [20,21] and modified to align with this study’s aim. Pilot testing was conducted with two pharmacists, whose feedback and experiences were instrumental in refining the questionnaire and in deciding to use a mixed-methods study design. Additionally, one pharmacist served as an expert reviewer, providing critical insights that further improved the questionnaire items. These items included (1) pharmacists’ demographics (multiple-choice questions), (2) barriers to accessing community pharmacy services by disabled persons (accessibility, designated parking spots, priority services, and pharmacy training in caring for this population) (Likert-scale), (3) perceived difficulty in patient education for people with hearing, visual, and learning disabilities (Likert-scale), and (4) pharmacists’ recommendations for optimizing care for this population (open-ended question). Consent was obtained prior to accessing the electronic survey. Participant confidentiality was rigorously maintained throughout this study by assigning numbers to each participant, ensuring that personal identifiers were not linked to the data. All the collected data were stored securely in encrypted files accessible only to the researcher. Additionally, any published results were presented in their aggregate form, with no individual responses or identifying details disclosed, further safeguarding participant anonymity. The questionnaire items are provided in Appendix A.

### 2.4. Data Analysis

Quantitative data were recorded and analyzed using SPSS version 20. Categorical data were summarized using frequencies and percentages, while continuous variables were described using mode, median, and frequency. Open-ended questions were analyzed through the thematic analysis of pharmacists’ recommendations. The process began with familiarization, where data was reviewed to understand its content. Initial codes were generated by identifying key segments, which were then grouped into broader themes based on patterns. These themes were reviewed and refined to ensure accuracy. Finally, the themes were clearly defined and organized into a narrative, supported by direct quotes. Microsoft Excel was used to manage and organize the analysis.

## 3. Results

### 3.1. Pharmacists’ Demographics and Experience Caring for Patients with Disabilities

A total of 40 community pharmacists completed the anonymized electronic survey. The majority was female (57.5%), between the age of 20 and 30 years old (60%), and worked at neighborhood pharmacies (55%). Most encountered disabilities were physical (67.5%) and hearing (60%) disabilities. To a lesser extent, visual (27.5%) and learning (25%) disabilities were also reported. Additionally, more than half of the pharmacists (57.5%) experienced some difficulty when caring for patients with disabilities, and most of them (65%) stated that there is a lack of specialized services for people with disabilities at the community pharmacies where they work (Table 1 and Table 2).

### 3.2. Community Pharmacy Services’ Ease of Access (Median, Mode, and Frequency)

When pharmacists were asked to rank barriers in pharmacy service access encountered by the disabled population, using a Likert-scale of (1) no barrier at all to (5) extreme barriers, the majority felt that there are some barriers in community pharmacy accessibility (parking, access ramps, wide aisles, escalators, elevators, etc.) (median: 3, mode: 3, frequency: 2.73) and in prioritizing disabled patients (median: 3, mode: 1, frequency: 2.8). The availability of trained pharmacy staff assisting disabled persons was the greatest obstacle perceived (median: 3, mode: 3, frequency: 3.5) (Table 3).

### 3.3. Pharmacists’ Perceived Difficulty in Educating Patients with Hearing, Visual, and Learning Disabilities (Median, Mode, and Frequency)

Pharmacists reported difficulties in educating patients with all visual (median: 3, mode: 5, frequency: 3.68), hearing (median: 4, mode: 4, frequency: 3.4), and learning disabilities (median: 3, mode: 3, frequency: 3.48) (Table 3).

### 3.4. Thematic Analysis on Community Pharmacists’ Recommendations to Improve Communication and Patients with Various Disabilities

This part of the questionnaire encouraged pharmacists to write their recommendations. Three major themes were identified for community pharmacists’ recommendations on ways to ease communication, optimize care, and ensure equality across all patients. These include (3.4.1) pharmacist training and awareness, (3.4.2) technology-guided methods to overcome communication barriers, and (3.4.3) better overall pharmacy accessibility.

#### 3.4.1. Pharmacist Training and Awareness

Most community pharmacists emphasized the need for training and educating pharmacists on the appropriate ways to assist patients with various disabilities. Summarized in Table 4. The following are some examples:

- *“Train pharmacy staff via intensive courses to assure health equality”* (Participant #2, #12, #13, #16, #26, #28, #32, and #37).
- *“Educate all pharmacy staff on how to appropriately handle people with disability of all types.”* (Participant #28, and #30).
- *“…We need compulsory courses to help understand this population’s [people with disabilities] needs and requirements”* (Participant #36).
- *“Ensure the availability of at least one trained personnel who can serve patients with different sorts of disabilities.”* (Participant #16).
- *“Having a trained pharmacist who can communicate in sign language can improve our service.”* (Participant #26).

Other community pharmacists advocated for awareness campaigns targeting health equality across patients with health disparities in general, including patients with disabilities. They also called for prioritizing patients with disabilities, as they require more time to ensure proper consultation.

- *“We need awareness campaigns to normalize prioritization of patients with disabilities.”* (Participant #33).
- *“Community awareness to prioritize patients with disabilities and those in need of special care.”* (Participant #22).
- *“Priority lane for patients with disabilities and assuring privacy when discussing their medications.”* (Participant #11).
- *“Prioritize patients with disabilities.”* (Participant #37).

#### 3.4.2. Technology-Guided Methods to Overcome Communication Barriers

Community pharmacists also recommended the use of various technological methods to assist in communicating with patients with visual, learning, and hearing disabilities, summarized in Table 4. The following are some examples:

- *“Having a play screen with visual and audio effects to educate visually or hearing-impaired patients can be beneficial.”* (Participant #1);
- *“Provide apps [mobile applications] to support patient counseling for hearing and visually disabled patients.”* (Participant #5);
- *“We should use assistive applications, pictures and brochures.”* (Participant #7, #24, #25, and #40);
- *“Create specialized applications to be used by the disabled population to help them communicate with healthcare providers.”* (Participant #8);
- *“Use barcodes in the form of stickers that can be placed on patient’s medication boxes or bottles to help provide them with the patient education they need in the form they may require, such as audio or visual material.”* (Participant #9 and #34);
- *“Provide a QR code that directs patients to follow specific instructions for taking certain medication, specifically for inhalers, nasal sprays, suppositories and injectables. This can be used for both patients with hearing and visual disabilities!”* (Participant #14).

One pharmacist suggested establishing a trusted governmental authority that can be accessed via telehealth video communication by all pharmacies and healthcare systems across the country to assist the hearing-disabled population by communicating and translating medical-specific sign language. This method could be a safer and more effective alternative to training pharmacists on sign language.

- *“Having a competent, trusted person assigned by the authority can help greatly in communicating [via telehealth] with patients in sign language. The person specialized in medical-specific sign language can sure be relied on for patient education, taking patient history, recommending an OTC medication, etc. This can be the link between the patient and anyone in the healthcare system to assure safe and effective translation, not just pharmacists.”* (Participant #33).

Other recommended technical methods help with communication, including the use of (1) printed patient education material (with or without visual aids), (2) photographed pictures, and (3) braille language on medication boxes. The following are some examples:

- *“Provide patients with printed educational materials in patient’s language, whether Arabic, English or braille.”* (Participant #23).
- *“Take pictures for the different steps used with certain medications, such as inhalers, and then print them out for patients who have hearing disability”* (Participant #14).
- *“The use of braille language as a sticker that can be added on the medication box to help the patient remember medication frequency and what it is used for.”* (Participant #34).

#### 3.4.3. Better Overall Pharmacy Accessibility

Nevertheless, some community pharmacists called for better overall pharmacy accessibility, as well as the improved availability of specialized counters that are designed for patients with disabilities and are specifically tailored to accommodate their various needs. These counters typically feature adjustable heights to ensure comfortable use by individuals in wheelchairs or those with mobility impairments. They may include accessible design elements such as wide space for maneuvering, low-height counters for easy reaching, and clear, easy-to-read signage in large fonts or braille. The following are some examples:

- *“Special counters for them [patients with disabilities] used for patient education and medication review”* (Participant #10).
- *“The pharmacy should be easily accessible for patients with physical disabilities.”* (Participant #17).
- *“Add more signs in the pharmacy to help navigate patients with disabilities around the pharmacy”* (Participant #34).

## 4. Discussion

The Social Model of Disability emphasizes that disability is not merely a result of an individual’s impairment but is significantly shaped by the barriers and limitations imposed by society. According to this model, the challenges faced by individuals with disabilities are due to environmental, organizational, and attitudinal barriers, rather than the impairments themselves [22]. Shifting the focus from individual impairments to addressing and removing societal obstacles creates a more equitable healthcare environment.

This study is groundbreaking, as it represents the first investigation into the barriers faced by community pharmacists when serving patients with disabilities in Saudi Arabia. The findings of this study highlight several significant barriers that these pharmacists encounter, which can profoundly impact the quality of care and access to pharmaceutical services for individuals with disabilities.

The identified barriers, such as inadequate pharmacist training and limited specialized services, can adversely affect patient outcomes. Pharmacists lacking training in disability-specific needs may struggle to provide appropriate medication management, potentially leading to medication errors, non-adherence, and suboptimal health outcomes [23]. Inadequate patient education, particularly for those with visual, hearing, or physical impairments, can result in poor understanding of medication instructions and potential health risks, thereby exacerbating existing health issues [24].

Furthermore, the economic implications of these barriers are substantial. Ineffective medication management and poor patient outcomes can increase healthcare costs due to more frequent medical visits, hospitalizations, and treatments for complications [25]. Additionally, the lack of accessible services may lead to reduced productivity and increased caregiving costs for patients and their families [26]. Investing in training and accessible services could mitigate these costs by enhancing medication adherence and reducing the incidence of avoidable health complications [27].

Understanding and addressing these challenges are crucial steps toward creating a more inclusive healthcare system. Over half of pharmacists in this study experienced difficulties when caring for patients with disabilities, and most indicated a lack of specialized services for these patients at their community pharmacies.

One of the primary barriers identified in this study is the lack of pharmacist training and awareness. Currently, the 2019 update of the American College of Clinical Pharmacy Pharmacotherapy Didactic Curriculum Toolkit does not include courses or topics that raise awareness about how this population should be educated with regard to their medications [28]. Moreover, there is no specific training within community pharmacies on serving this population. Pharmacy schools should prepare their graduates to meet the diverse needs of different populations and address potential communication barriers. Insufficient knowledge and awareness among community pharmacists regarding disabilities pose a significant challenge. Understanding the unique medication management needs, assistive devices, and available support services for patients with disabilities is essential to providing appropriate care. Integrating disability-related education into pharmacy curricula and offering continuing education programs can help bridge this knowledge gap and empower pharmacists to deliver tailored services to patients with disabilities.

Community pharmacists also encountered difficulties in effectively educating patients with disabilities, particularly those with visual, hearing, and physical impairments. The diverse nature of these disabilities often requires tailored communication methods, as traditional approaches may not be suitable or effective for all individuals. Consequently, innovative solutions are needed to address this communication gap and ensure comprehensive patient education.

Play screens with visual and audio effects can enhance patient understanding by providing interactive educational materials that cater to different learning styles. These screens can present medication instructions, health information, and drug interactions in a visually engaging manner, accompanied by audio explanations for individuals with hearing impairments [29].

Mobile phone applications offer a convenient platform for patient education. These applications can provide medication reminders, drug information, and access to virtual consultations with pharmacists. By delivering personalized and accessible content, mobile applications empower patients with disabilities to actively participate in their healthcare management [24].

The use of QR codes in patient education is another innovative approach. Pharmacists can provide QR codes on medication labels or educational materials which patients can scan with their smartphones to access additional information, instructions, or video tutorials. This simple and user-friendly method accommodates individuals with various disabilities and promotes independent learning [30,31].

Finally, the increasing prominence of artificial intelligence (AI) presents promising opportunities for enhancing patient engagement in community pharmacy settings. AI-powered chatbots and virtual assistants can provide instant support, answering patients’ questions and addressing their concerns. Natural language processing algorithms can enable these AI systems to understand and respond to patients with different disabilities, fostering effective communication and enhancing patient education [25,26,27].

Another key recommendation from community pharmacists is the establishment of specialized locations within pharmacies designed specifically to cater to the needs of patients with disabilities. These dedicated spaces would provide a suitable environment for educating and serving these patients, ensuring that their unique requirements are met. Creating designated areas equipped with appropriate resources and assistive devices can enhance accessibility and facilitate better pharmaceutical care for individuals with disabilities. Also, the addition of more signs in the pharmacy is another crucial aspect emphasized by community pharmacists [31]. Clear signage plays a significant role in improving accessibility and wayfinding for patients with disabilities. These signs can indicate accessible routes, service counters, medication sections, and other relevant areas within the pharmacy.

The suggestions put forward by pharmacists align with the recommendations outlined in the American National Council for Disability (NCD) report, which underscores the health disparities and barriers faced by individuals with disabilities due to architectural and programmatic accessibility barriers [7]. By implementing specialized locations, improving signage, establishing priority lanes, and setting up specialized counters, community pharmacies can take significant steps toward fostering a more inclusive and accessible environment.

To address these barriers, collaborative efforts are needed from various stakeholders. Government agencies, regulatory bodies, and pharmacy associations should prioritize the development and implementation of policies and guidelines that promote inclusivity in community pharmacies. Collaborations with disability organizations and experts can provide valuable insights and help shape effective strategies for overcoming barriers. While this study focused specifically on the barriers faced by community pharmacists in Saudi Arabia, the findings can be extrapolated to other contexts as well. Improving the accessibility and inclusivity of community pharmacies is a global issue that requires attention and action.

This cross-sectional study has certain limitations that need to be acknowledged. Firstly, the small sample size and convenience sampling method restrict the generalizability of the findings to a larger population and introduce potential selection bias. However, despite the limited sample, this study managed to capture diverse perspectives and generate rich data which can serve as a valuable foundation for establishing standards and policies in serving patients with disabilities.

While this study provides valuable insights, the findings are specific to community pharmacies in Saudi Arabia and may not fully apply to other contexts due to differences in healthcare systems, training programs, and cultural factors. Acknowledging these limitations, we suggest that similar studies be conducted in different settings to validate and expand upon our findings.

It is important to note that this study did not assess patients with mixed disabilities, which may present unique challenges for pharmacists in providing optimal care. Future research should focus on exploring the specific needs and barriers faced by this population to develop tailored strategies and interventions that address their diverse requirements.

While this study highlights the potential of technological solutions in improving patient outcomes, further research is necessary to evaluate their effectiveness, usability, and impact on healthcare delivery. Robust studies with larger sample sizes and controlled designs are needed to determine the true benefits of these technological interventions in community pharmacy settings.

Moreover, future studies should investigate the integration of artificial intelligence (AI) technologies, such as machine learning and predictive analytics, in community pharmacies. These AI-powered systems have the potential to enhance patient education, medication adherence, and overall health outcomes. Conducting research in this area will provide valuable insights into the efficacy and feasibility of implementing AI technologies in community pharmacy practice.

Despite these limitations, the findings contribute to existing knowledge and can inform the development of standards and policies for serving patients with disabilities. The mixed-methods approach strengthened this study by allowing for a comprehensive analysis of both quantitative and qualitative data. Including pharmacists’ perspectives provides a nuanced understanding of the barriers and potential solutions from those directly involved in patient care. This approach enriches the findings and supports the development of targeted interventions.

## 5. Conclusions

In conclusion, this study has identified several critical barriers that community pharmacists in Saudi Arabia face when serving patients with disabilities. The most significant barriers include a lack of specialized training and awareness, communication challenges with patients who have various disabilities, and the inadequacy of physical environments in community pharmacies to accommodate these patients. To address these barriers, this study recommends the following: (1) Enhancing pharmacist training: integrate disability-related content into pharmacy curricula and provide ongoing professional development to ensure pharmacists are equipped to meet the needs of patients with disabilities. (2) Improving communication methods: adopt innovative communication tools, such as interactive screens, mobile applications, and QR codes, to better educate and engage patients with disabilities. (3) Creating accessible pharmacy environments: establish specialized areas within pharmacies and improve signage to enhance accessibility for patients with disabilities.

These recommendations aim to foster a more inclusive and equitable healthcare environment, ultimately improving the quality of care and health outcomes for individuals with disabilities. These efforts will lead to better medication adherence, reduced healthcare costs, and an overall improvement in the quality of life for patients with disabilities.

Furthermore, the integration of technology and AI in pharmacy practices has the potential to revolutionize patient engagement and care, ensuring that community pharmacies become more inclusive and patient-centered over time. Further research in this field is crucial to evaluate the effectiveness, sustainability, and potential benefits of these solutions.

## Figures and Tables

**Table 1:** Pharmacists’ and pharmacies’ demographics (*n* = 40).

Variable		N (%)
**Gender**	Male	17 (42.5)
	Female	23 (57.5)
**Age Group**	20 to 30 years old	24 (60)
	31 to 40 years old	12 (30)
	41 to 50 years old	2 (5)
	>50 years old	2 (5)
**Years of experience**	1 to 3 years	19 (47.5)
	4 to 6 years	3(7.5)
	7 to 9 years	9 (22.5)
	>10 years	9 (22.5)
**Pharmacy location**	Neighborhood pharmacy	22 (55)
	Within a shopping center	5 (12.5)
	Within a healthcare center	13 (32.5)
**Encountered disability**	Physical disability	27 (67.5)
	Hearing disability	24 (60)
	Visual disability	11 (27.5)
	Learning disability	10 (25)
**Difficulty in caring for patients with disabilities**	Difficult	13 (32.5)
	Somewhat difficult	10 (25%)
	Not difficult	17 (42.5)
**Availability of specialized services for people with disabilities at the community pharmacy**	Available	7 (17.5)
	Somewhat available	7 (17.5)
	Not available	26 (65)

**Table 2:** Participant’s specific demographics (*n* = 40).

Participant #	Age Range	Sex	Experience (Years)	Title	Pharmacy Location
Participant 1	31–40	Female	4–6	Pharmacist	Within a shopping center
Participant 2	31–40	Female	>10	Pharmacist	Neighborhood pharmacy
Participant 3	20–30	Female	1–3	Pharmacist	Within a shopping center
Participant 4	20–30	Male	7–9	Pharmacist	Neighborhood pharmacy
Participant 5	20–30	Female	1–3	Pharmacy intern	Neighborhood pharmacy
Participant 6	31–40	Male	1–3	Pharmacy technician	Neighborhood pharmacy
Participant 7	20–30	Female	1–3	Pharmacy intern	Within a shopping center
Participant 8	20–30	Female	1–3	Pharmacy intern	Within a health center
Participant 9	20–30	Female	1–3	Pharmacist	Within a health center
Participant 10	20–30	Female	1–3	Pharmacy resident	Within a health center
Participant 11	31–40	Female	>10	Pharmacy technician	Within a health center
Participant 12	20–30	Female	1–3	Pharmacist	Within a health center
Participant 13	20–30	Female	1–3	Pharmacist	Within a health center
Participant 14	20–30	Male	1–3	Pharmacist	Within a health center
Participant 15	20–30	Female	1–3	Pharmacist	Neighborhood pharmacy
Participant 16	20–30	Male	1–3	Pharmacist	Within a health center
Participant 17	31–40	Female	7–9	Pharmacy technician	Within a health center
Participant 18	31–40	Male	>10	Pharmacist	Neighborhood pharmacy
Participant 19	20–30	Male	7–9	Pharmacist	Neighborhood pharmacy
Participant 20	20–30	Female	1–3	Pharmacist	Within a shopping center
Participant 21	>50	Male	>10	Pharmacist	Neighborhood pharmacy
Participant 22	20–30	Male	7–9	Pharmacist	Neighborhood pharmacy
Participant 23	41–50	Female	>10	Pharmacist	Neighborhood pharmacy
Participant 24	20–30	Female	1–3	Pharmacist	Neighborhood pharmacy
Participant 25	31–40	Female	7–9	Pharmacist	Within a health center
Participant 26	20–30	Female	1–3	Pharmacist	Neighborhood pharmacy
Participant 27	31–40	Female	7–9	Pharmacy resident	Within a health center
Participant 28	20–30	Male	1–3	Pharmacist	Neighborhood pharmacy
Participant 29	41–50	Male	>10	Pharmacist	Neighborhood pharmacy
Participant 30	20–30	Female	1–3	Pharmacist	Within a health center
Participant 31	20–30	Female	1–3	Clinical pharmacist	Neighborhood pharmacy
Participant 32	31–40	Male	>10	Pharmacist	Neighborhood pharmacy
Participant 33	31–40	Male	>10	Pharmacist	Neighborhood pharmacy
Participant 34	20–30	Female	1–3	Pharmacy intern	Within a shopping center
Participant 35	20–30	Male	4–6	Clinical pharmacist	Neighborhood pharmacy
Participant 36	20–30	Male	4–6	Pharmacist	Neighborhood pharmacy
Participant 37	31–40	Male	>10	Pharmacist	Neighborhood pharmacy
Participant 38	>50	Male	7–9	Pharmacist	Neighborhood pharmacy
Participant 39	20–30	Male	7–9	Pharmacist	Neighborhood pharmacy
Participant 40	31–40	Female	7–9	Clinical pharmacist	Within a health center

**Table 3:** Community pharmacy services’ ease of access for patients with disabilities (*n* = 40).

	Median *	Mode *	Frequency *
Barriers in accessing community pharmacy services			
Accessibility to community pharmacy (parking, access ramps, wide aisles, escalators, elevators, etc.)	3	3	2.73
Prioritizing patients with disabilities	3	1	2.8
Availability of a pharmacy staff trained in communicating and serving patients with disabilities	3	3	3.5
Perceived difficulty in educating patients with:			
Visual disability	4	5	3.68
Hearing disability	4	4	3.4
Learning disability	3	3	3.48

* Community pharmacists ranked these barriers from (1) no barrier at all, (2) a few barriers, (3) some barriers, (4) moderate barriers, and (5) extreme barriers. The median is the average number of data, the mode is the value that appeared most frequently in the data, and the frequency is the number of times the value occurred in the data.

**Table 4:** Recommended technology-guided methods for assisting patients with visual, learning, and hearing disabilities.

Technology/Method	Description	Recommended By
**Play Screens**	Screens with visual and audio effects to aid communication and education for patients with visual or hearing impairments.	Participant #1
**Mobile Application**	Applications designed to support patient counseling for hearing and visual disabilities.	Participant #5
**Assistive Applications**	Applications that include pictures, brochures, and other assistive features.	Participants #7, #24, #25, #40
**Specialized Applications**	Custom apps for patients with disabilities to facilitate communication with healthcare providers.	Participant #8
**QR Codes**	QR codes on medication boxes or bottles to provide patient education in various formats (audio, visual).	Participants #9, #34, #14
**Telehealth Video Communication**	A trusted governmental authority providing sign language translation via telehealth for improved communication with hearing-impaired patients.	Participant #33
**Printed materials**	Educational materials in different languages, including braille, for patient education.	Participant #23
**Photographed Instructions**	Pictures depicting medication use steps, especially for hearing-impaired patients.	Participant #14
**Braille Labels**	Braille stickers on medication boxes to indicate medication frequency and purpose.	Participant #34

## Data Availability

The datasets used and/or analyzed during the current study are available from the corresponding author on reasonable request.

## Appendix A

**Table A1:** Questionnaire Items.

Section	Question	Possible Answer(s)
**1- Pharmacist demographic**	Sex	Male Female
	Age group	20–23 years old 31–40 years old 41–50 years old >50 years old
	Years of experience	1–3 years 4–6 years 7–9 years >10 years
	Professional classification	Pharmacist Pharmacy intern Pharmacy resident Pharmacy technician Pharmacy consultant and/or clinical pharmacist
	Residential region	Western region Central region Eastern region Northern region Southern region
	Pharmacy location	Neighborhood pharmacy Within a shopping center Within a health center
**2- Pharmacist Opinion Towards the Community Pharmacy Services for Persons with Disabilities**	1. During your work in the pharmacy, did you encounter any person with disability?	Yes No Maybe
	2. What type of disability did you encounter? (Can mark more than one)	Visual Hearing Learning Physical None
	3. Did you have difficulty educating disabled persons on their medication?	Yes No Maybe
	4. Are there any specialized services for persons with disabilities being provided in your community pharmacy?	Yes No Maybe
**3- Perceived barriers to Accessing Community Pharmacy Services for Disabled Persons**	1. Accessibility to community pharmacy (slide, handles, etc.)	5 = Extreme barriers, 4 = moderate barriers, 3 = some barriers, 2 = few barriers, 1 = no barriers at all
	2. Designated parking spots for disabled persons	
	3. Priority for serving patients with disabilities	
	4. Communicating and serving a disabled person	
	5. Educating patients with visual disability (ex: providing audio material)	
	6. Educating patients with hearing disability (ex: providing visual and/or written material)	
	7. Educating patients with learning disability (ex: reaching out for family members)	
**4- Adjustments Needed in Community Pharmacies for Persons with Disabilities**	1. What would you recommend for effective communication and patient education for people of various disabilities?	○ Short answer
	2. Feel free to add any additional comments	○ Short answer
